# Systematic Genetic Interaction Analysis Identifies a Transcription Factor Circuit Required for Oropharyngeal Candidiasis

**DOI:** 10.1128/mbio.03447-21

**Published:** 2022-01-11

**Authors:** Norma V. Solis, Rohan S. Wakade, Virginia E. Glazier, Tomye L. Ollinger, Melanie Wellington, Aaron P. Mitchell, Scott G. Filler, Damian J. Krysan

**Affiliations:** a Division of Infectious Diseases, Lundquist Institute for Biomedical Innovation at Harbor-UCLA Medical Center, Torrance, California, USA; b Department of Pediatrics, Carver College of Medicine, University of Iowa grid.412584.egrid.214572.7 , Iowa City, Iowa, USA; c Department of Biology, Niagara University, Niagara Falls, New York, USA; d Department of Microbiology, University of Georgia, Athens, Georgia, USA; e Department of Medicine, David Geffen School of Medicine at UCLA, Los Angeles, California, USA; f Department of Microbiology and Immunology, Carver College of Medicine, University of Iowa grid.412584.egrid.214572.7 , Iowa City, Iowa, USA; Geisel School of Medicine at Dartmouth

**Keywords:** *Candida albicans*, biofilms, complex haploinsufficiency, oropharyngeal candidiasis

## Abstract

Oropharyngeal candidiasis (OPC) is a common infection that complicates a wide range of medical conditions and can cause either mild or severe disease depending on the patient. The pathobiology of OPC shares many features with candidal biofilms of abiotic surfaces. The transcriptional regulation of C. albicans biofilm formation on abiotic surfaces has been extensively characterized and involves six key transcription factors (Efg1, Ndt80, Rob1, Bcr1, Brg1, and Tec1). To determine if the *in vitro* biofilm transcriptional regulatory network also plays a role in OPC, we carried out a systematic genetic interaction analysis in a mouse model of C. albicans OPC. Whereas each of the six transcription factors are required for *in vitro* biofilm formation, only three homozygous deletion mutants (*tec1*ΔΔ, *bcr1*ΔΔ, and *rob1*ΔΔ) and one heterozygous mutant (*tec1*Δ/*TEC1*) have reduced infectivity in the mouse model of OPC. Although single mutants (heterozygous or homozygous) of *BRG1* and *EFG1* have no effect on fungal burden, double heterozygous and homozygous mutants have dramatically reduced infectivity, indicating a critical genetic interaction between these two transcription factors during OPC. Using epistasis analysis, we have formulated a genetic circuit, [*EFG1*+*BRG1*]→*TEC1*→*BCR1*, that is required for OPC infectivity and oral epithelial cell endocytosis. Surprisingly, we also found transcription factor mutants with *in vitro* defects in filamentation, such as *efg1*ΔΔ, *rob1*ΔΔ, and *brg1*ΔΔ filament, during oral infection and that reduced filamentation does not correlate with infectivity. Taken together, these data indicate that key *in vitro* biofilm transcription factors are involved in OPC but that the network characteristics and functional connections during infection are distinct from those observed *in vivo*.

## INTRODUCTION

Candida albicans is a commensal component of the human mycobiome that frequently causes mucosal infections of both immunocompetent and immunocompromised people (1). The oral cavity is an important C. albicans niche and, consequently, this organism causes both mucosal and dental infections under specific conditions (2). The infections of the oral mucosa caused by C. albicans range from minor, superficial infections such as neonatal thrush to extensive pharyngeal and/or esophageal infections that cause significant morbidity. Oropharyngeal candidiasis (OPC) complicates a variety of medical conditions, including diabetes, radiation therapy for head and neck cancers, and alterations in saliva production (3). The most severe infections occur in patients who have altered T-cell immunity. For example, people living with HIV still frequently developed OPC in the era of highly effective retroviral therapy (4). In addition, specific primary immune deficiencies such as chronic mucocutaneous candidiasis and others lead to persistent OPC (5). Furthermore, the development of immunomodulating therapies in other areas of medicine continues at a brisk pace, leading to increasing numbers of patients who are at risk for fungal diseases such as OPC. Therefore, understanding the pathobiology of this relatively common fungal infection is not only of fundamental importance to fungal pathogenesis but also could inform the development of innovative approaches to its treatment.

A cardinal clinical feature of OPC is the development of tissue-adherent plaques of yeast and hypha-stage fungi (2, 3). Histological analysis of these lesions is consistent with C. albicans adopting features of biofilm-phase growth, such as surface adherent replication and extracellular matrix formation (6). Results from previous investigations, including those from our groups, have supported this general model at both the cellular and molecular level. For example, the transcriptional regulator of *in vitro* biofilm formation, Bcr1 (7), is also required for infection and virulence in a mouse model of OPC (8). Large-scale, systematic genetic studies have identified a set of C. albicans transcription factors (TF) that are required for *in vitro* biofilm formation (9, 10). Recently, we have also used a complex haploinsufficiency-based approach to identify key interactions between the TFs of this network during different stages of *in vitro* biofilm formation (11, 12).

To further explore the overlap between the regulation of *in vitro* biofilm formation and oral infection, we asked which members of the *in vitro* biofilm transcriptional regulatory network (*TEC1*, *EFG1*, *BRG1*, *NDT80*, *BCR1*, and *ROB1*) are required to establish oral infections (9). In addition, we used our array of 12 double heterozygous TF deletion strains to identify pairs of TFs critical for OPC (11). Our genetic data indicate that Efg1 and Brg1 are critical for activation of the Tec1-Bcr1 axis. We also provide evidence that this genetic circuit regulates the ability of C. albicans to invade oral epithelial cells.

## RESULTS

### The transcriptional network for establishment of OPC is distinct from *in vitro* biofilm formation.

**FIG 1:**
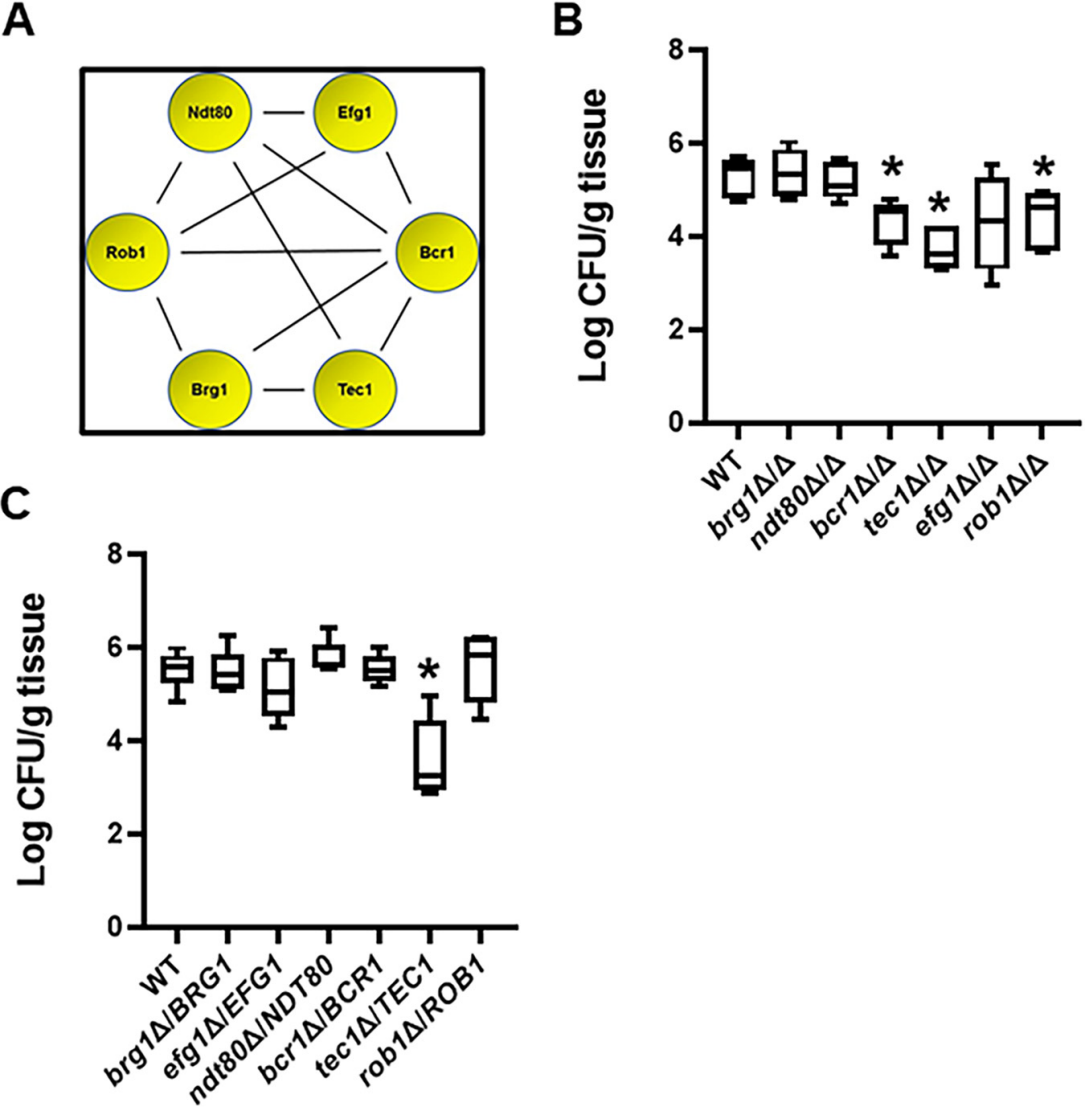
Effect of *in vitro* biofilm transcription factors in a mouse model of oropharyngeal candidiasis. (A) The functional genetic interaction network of six transcription factors required for C. albicans biofilm formation *in vitro* (11). (B and C) The fungal burden 5 days postinfection for animals infected with the indicated deletion mutants in the SN background. Medians and standard deviations are shown for 4 to 5 animals per group. The log_10_-transformed fungal burden data for each experiment was analyzed by one-way ANOVA followed by *post hoc* Student's *t* test to identify statistically significant differences between individual strains (*P* < 0.05). Strains that were statistically different from the WT are indicated with an asterisk.

In 2012, Nobile et al. characterized a network of six TFs (9) that are essential for the formation of biofilms *in vitro* (Fig. 1A). As part of these studies, they also showed that the TFs were, by and large, required for *in vivo* biofilm formation using rat models of vascular catheter and denture infections; *bcr1*ΔΔ showed a less pronounced effect in the denture model than in the catheter model. Both of the *in vivo* models studied by Nobile et al. involve the formation of a C. albicans biofilm on an inanimate surface within the host (9). As discussed above, OPC caused by C. albicans shows features of biofilm-stage growth (6). An OPC-related biofilm represents the adhesion to, and infection of, a mucosal surface rather than the abiotic surface studied by Nobile et al. (9).

To determine the role of *in vitro* biofilm TFs in a mouse model of OPC, we infected corticosteroid-treated outbred CD-1 mice with both homozygous and heterozygous deletion mutants of the six biofilm TFs. Tissue fungal burden was determined 5 days after inoculation. As shown in Fig. 1B, three homozygous deletion mutants, *tec1*ΔΔ, *rob1*ΔΔ, and *bcr1*ΔΔ, showed a reduction in tissue fungal burden; the *tec1*Δ/*TEC1* heterozygous mutant was the only strain to show simple haploinsufficiency (Fig. 1C). Bcr1 was previously shown to be required for OPC, but the roles of Tec1 and Rob1 had not been reported (8). Thus, a set of three *in vitro* biofilm-related transcription factors are required for OPC infectivity in a mouse model, whereas deletion mutants in three other transcriptional regulators (Ndt80, Efg1, and Brg1) of *in vitro* biofilms do not alter OPC infectivity. Importantly, Nobile et al. found that all six regulators were required to form normal biofilms in rodent models of intravascular catheter and denture biofilm formation (9), further emphasizing the notion that different TF networks and interactions are required for different types of C. albicans infections.

### Complex haploinsufficiency-based analysis reveals critical interaction between Brg1 and Efg1 during murine OPC.

**FIG 2:**
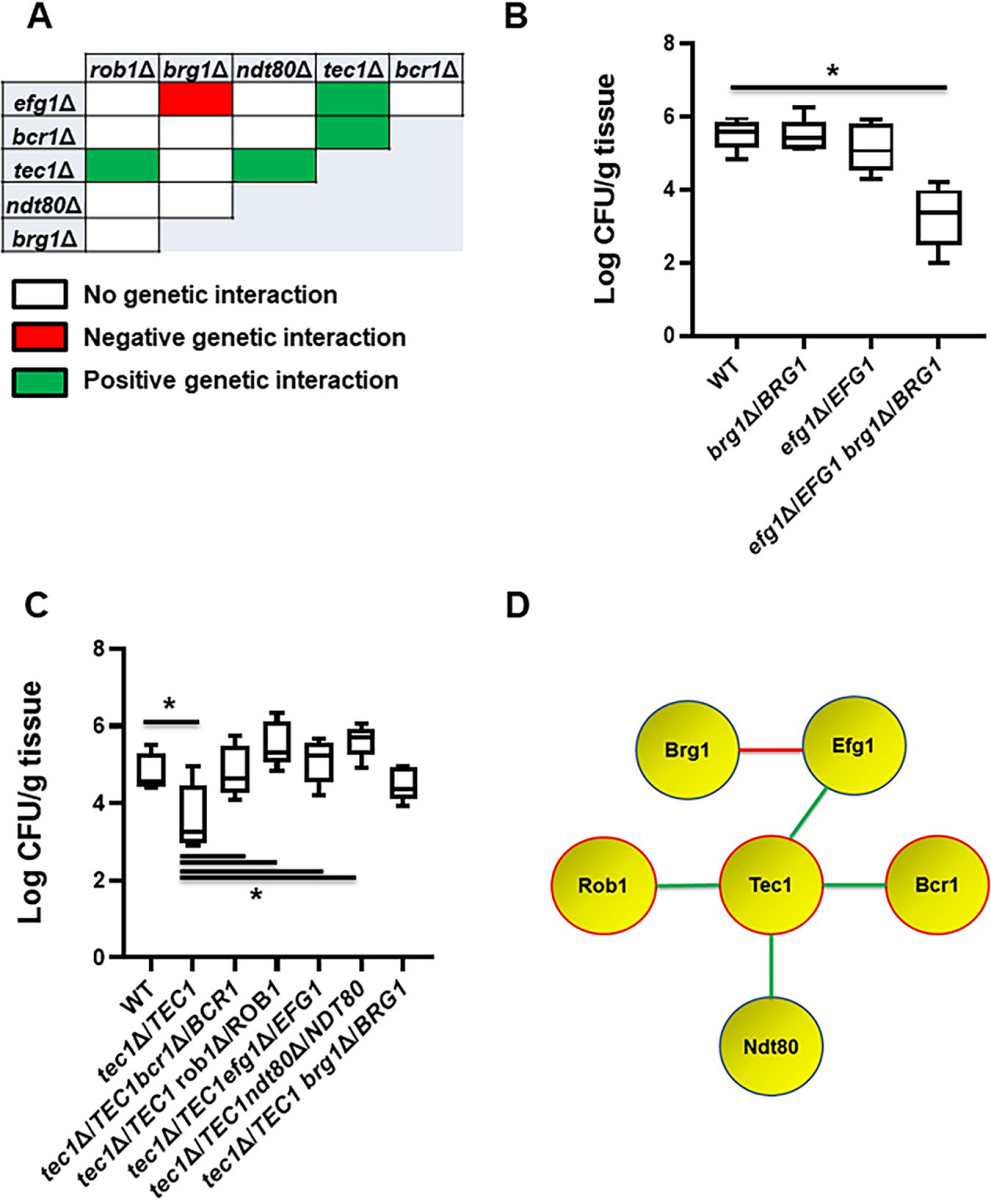
Genetic interaction analysis of transcription factors in a mouse model of oropharyngeal candidiasis identifies a functional network. (A) The set of all possible double heterozygous transcription factor deletion mutants was screened for genetic interactions relative to the individual heterozygotes. The interaction map summarizes these interactions with negative interactions indicated in red, positive interactions indicated by green, and no interaction indicated by white. (B) The double heterozygous *efg1*Δ/*EFG1 brg1*Δ/*BRG1* mutant shows complex haploinsufficiency in the oropharyngeal candidiasis model. The log_10_-transformed fungal burden data, means and standard deviations, for each experiment were analyzed by one-way ANOVA followed by *post hoc* Student's *t* test to identify statistically significant differences between individual strains (*P* < 0.05). Strains that were statistically different from the WT are indicated with an asterisk. (C) *BCR1*, *ROB1*, *NDT80*, and *EFG1* show positive genetic interactions with *tec1*Δ/*TEC1*. (D) The functional interaction network based on genetic interactions shown by the indicated transcription factors. Deletion mutants of genes highlighted in red have reduced infectivity. Red lines indicate a negative genetic interaction, while green lines indicate a positive genetic interaction.

Recently, we explored the genetic interactions between the *in vitro* biofilm TFs using a set of all possible double heterozygous mutants derived from the 6 core TFs (11). To identify genetic interactions during murine OPC, we used this set to examine the ability of the double heterozygous mutants to infect the oral mucosae compared to the corresponding single heterozygote and wild-type (WT) strains. In contrast to the six negative genetic interactions that were identified within this network during *in vitro* biofilm formation (11), only one negative interaction, between *brg1*Δ/*BRG1* and *efg1*Δ/*EFG1*, was identified (Fig. 2A and B). Interestingly, the reduction in fungal burden displayed by the *brg1*Δ/*BRG1 efg1*Δ/*EFG1* double heterozygote was similar to that shown by the *tec1*ΔΔ, *bcr1*ΔΔ, and *rob1*ΔΔ mutants. Furthermore, mice infected with the *brg1*Δ/*BRG1 efg1*Δ/*EFG1* mutant had lower fungal burden than animals infected with the homozygous deletion mutants of both *EFG1* and *BRG1*, further establishing the interdependence of the function of these two TFs during murine OPC.

In addition to the negative genetic interaction between *BRG1* and *EFG1*, we found that the haploinsufficiency of the *tec1*Δ/*TEC1* strain was suppressed in double heterozygotes containing *EFG1*, *NDT80*, *BCR1*, and *ROB1* deletions (Fig. 2A and C); the *tec1*Δ/*TEC1 brg1*Δ/*BRG1* mutant was not statistically different from the *tec1*Δ/*TEC1* single deletion, indicating no interaction. The most common reason for positive genetic interactions is that the two genes function in a linear pathway or trigger a compensatory response (13). For example, Tec1 is known to regulate Bcr1 based on *in vitro* studies (9); in addition, we have previously observed a positive genetic interaction between *tec1*Δ/*TEC1* and *bcr1*Δ/BCR1 during *in vitro* biofilm formation (11). Based on the data from Fig. 2B and C, the functional genetic interaction network for these TFs during OPC can be formulated as shown in Fig. 2D.

### Positive and negative genetic interactions observed during infection are reflected in expression of the OPC-related virulence gene *HWP1*.

**FIG 3:**
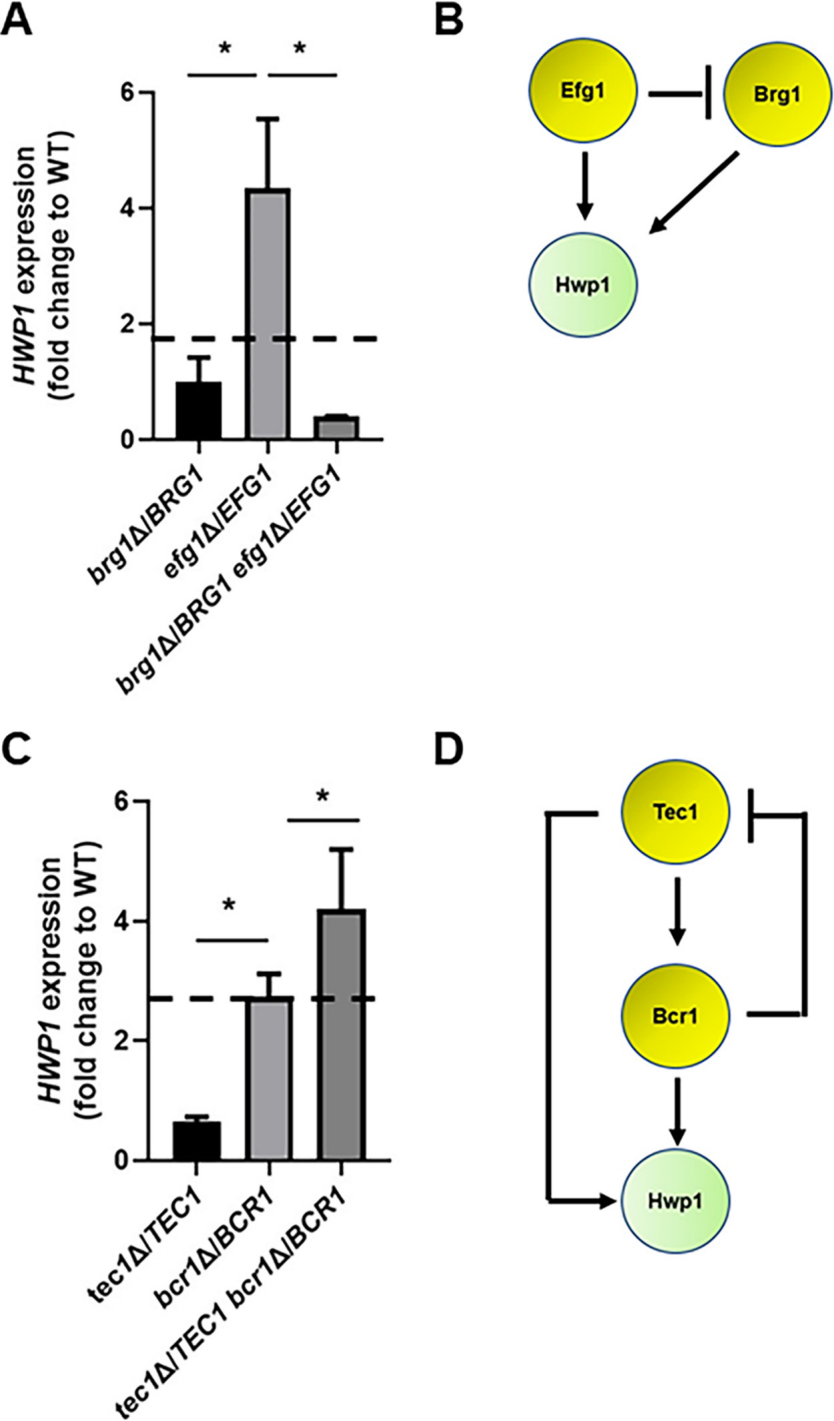
*HWP1* expression in double heterozygous deletion strains showing negative and positive genetic interactions. The expression of *HWP1* in the indicated strains in 48-h *in vitro* biofilms was assessed by RT-PCR as described in Materials and Methods. The dashed line indicates the fold change in *HWP1* expression if the two TFs functioned independently and thus displayed no genetic interaction. (A) The *brg1*Δ/*BRG1 efg1*Δ/*BRG1* strain shows a negative genetic interaction during OPC infection and a negative genetic interaction with respect to biofilm *HWP1* expression. (B) Regulatory circuit consistent with the effect of *TEC1* and *BCR1* mutations on *HWP1* expression. Arrows indicate activating interaction; cross bar indicates deactivating interaction. (C) The *tec1*Δ/*TEC1 brg1*Δ/*BRG1* strain shows a positive genetic interaction during OPC infection and a positive genetic interaction with respect to *HWP1* expression. (D) Regulatory circuit consistent with the effect of *BRG1* and *EFG1* mutations on *HWP1* expression. The data are presented as the means of 3 to 4 independent replicates performed in triplicate with standard errors of the means. Differences between mutants were analyzed by one-way ANOVA followed by Student's *t* test to assess significance of individual group differences (*P* < 0.05).

To further explore the two key interactions, suppression of *tec1*Δ/*TEC1* haploinsufficiency by other TF mutants and the negative interaction between *brg1*Δ/*BRG1* and *efg1*Δ/*EFG1*, we examined the effect of these mutants on the expression of *HWP1*. Hwp1 is a known target of Bcr1 during OPC, and its deletion leads to reduced fungal burden at infection day 5 (7), the time point used here. Unfortunately, technical limitations have prevented us from measuring *HWP1* expression reliably during infections with the mutants, likely due to reduced fungal burden for the key strains. As a surrogate, we used reverse transcription-PCR (RT-PCR) to measure *HWP1* expression during *in vitro* biofilm formation and transcriptionally characterize the negative interaction of *BRG1*-*EFG1* and the positive interaction of *TEC1*-*BCR1*. The single heterozygous *BRG1* deletion strain has near wild-type levels of *HWP1* expression while the *efg1*Δ/*EFG1* mutant has significantly increased levels of *HWP1* expression, suggesting a compensatory response to reduction in *EFG1* copy number (Fig. 3A). We used the multiplicative model for epistasis to formally analyze the effect of the *BRG1*-*EFG1* double mutant on *HWP1* expression as described in Materials and Methods and summarized in Fig. S1 in the supplemental material. The expected level of *HWP1* expression if Brg1 and Efg1 functioned independently (no genetic interaction) is shown in Fig. 3A, and the actual expression is ∼10-fold lower, consistent with a strong negative genetic interaction. A regulatory circuit that is consistent with these interactions is shown in Fig. 3B. Based on this circuit, reduction in *EFG1* copy number appears to increase Brg1 activity, which in turn leads to increased *HWP1* expression, potentially due to an imbalance in the interacting factors. Loss of one copy of *BRG1* then interrupts this compensatory response, leading to the dramatic reduction in *HWP1* expression.

10.1128/mbio.03447-21.1 FIG S1 Tables summarizing multiplicative model-based quantitative epistasis analysis of *BRG1*-*EFG1* and *TEC1*-*BCR1*. ε = [normalized phenotype of double mutant] – [expected phenotype of double mutant] where the expected phenotype is equal to the product of the normalized phenotypes of the individual single mutants. ε = 0 indicates no interaction and the genes function independently; ε > 1 indicates a positive or suppressive interaction; ε < 1 indicates a negative or aggravating interaction. (A) Analysis of fungal burden data. (B) Analysis of *HWP1* expression data. Download FIG S1, TIF file, 0.08 MB. Copyright © 2022 Solis et al. 2022 Solis et al. https://creativecommons.org/licenses/by/4.0/ This content is distributed under the terms of the Creative Commons Attribution 4.0 International license.

In the case of the positively interacting *TEC1*-*BCR1* pair, the *tec1*Δ/*TEC1* mutant has reduced expression of *HWP1* relative to the wild type, while the *bcr1*Δ/*BCR1* heterozygote has significantly increased expression (Fig. 3C). The increased expression of *HWP1* in the *bcr1*Δ/*BCR1* heterozygote suggests that *HWP1* is also increased in a compensatory response to a reduced *BCR1* copy number. In contrast to the case of the *BRG1*-*EFG1* interaction, the *tec1*Δ/*TEC1 bcr1*Δ/*BCR1* double mutant retains the elevated expression of the *bcr1*Δ/*BCR1* mutant and is consistent with Bcr1 functioning downstream of Tec1. These expression data are consistent with a more complex regulatory circuit involving Tec1 and Bcr1 (Fig. 3D). The opposite effects of reduced *TEC1* and *BCR1* copy number indicate a feedforward circuit in which Tec1 regulates *HWP1* expression directly and indirectly; the reduction of *TEC1* copy number seems to have a more significant effect on its direct activation of *HWP1*. Reduction in *BCR1* copy number leads to increased *HWP1* expression through a compensatory response that is consistent with feedback inhibition of Tec1. Relief of this inhibition is sufficient to compensate for a reduction in the copy number of both *TEC1* and *BCR1*. It is important to keep in mind that these are formal circuits that are consistent with the observed effects of the mutations on *HWP1* expression and the fact that the *HWP1* promoter is bound by Tec1, Bcr1, Efg1, and Brg1. Although the TFs in these circuits regulate each other’s expression, it is also possible that the effects are mediated through multi-TF complexes. Nonetheless, these data provide strong supporting evidence that the same type of genetic interactions observed for *in vivo* infection are also evident in a relevant *in vitro* phenotype.

### The complex haploinsufficient phenotype displayed by the *brg1*Δ/*BRG1 efg1*Δ/*EFG1* mutant is specific to OPC infection.

**FIG 4:**
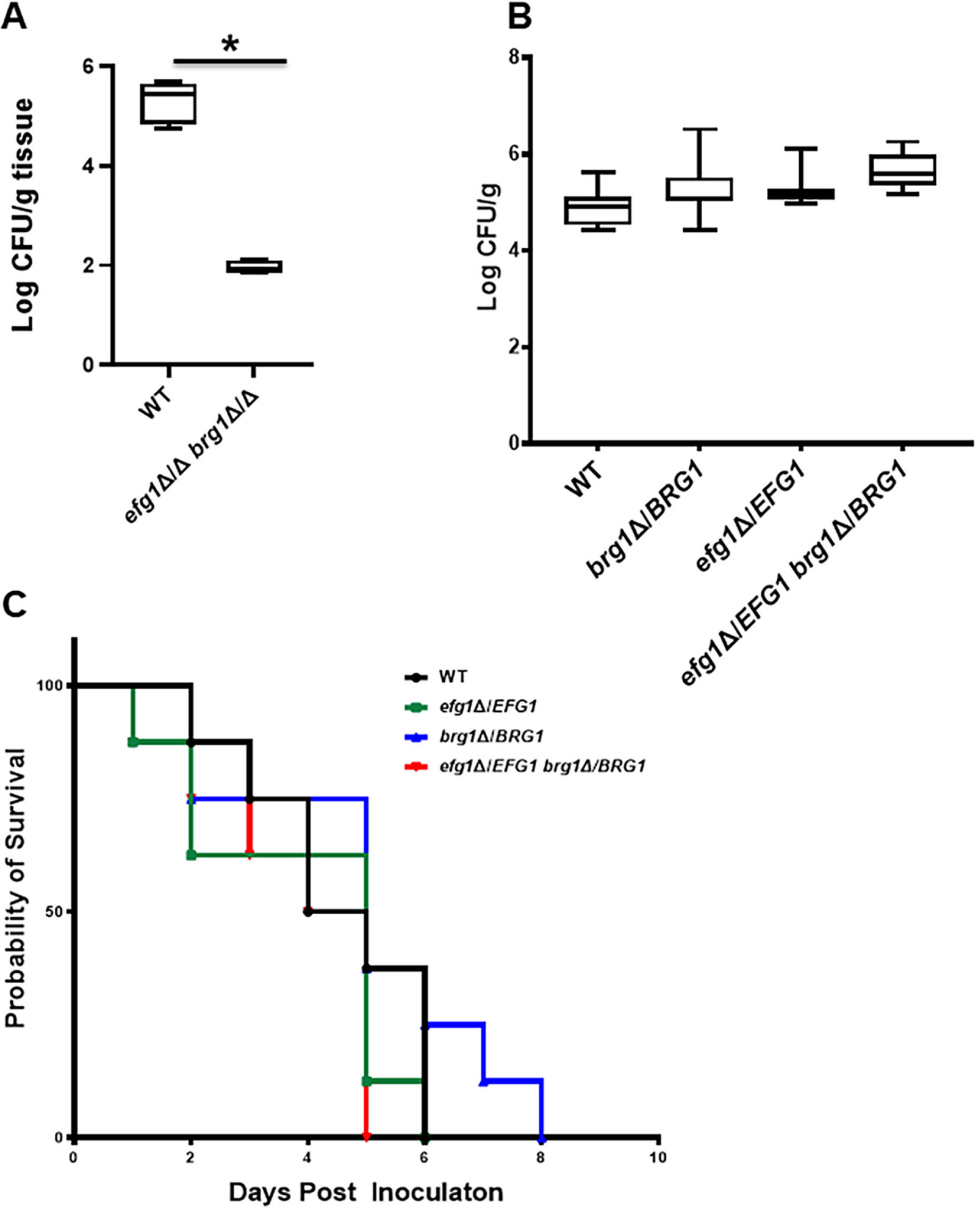
Genetic interaction of *EFG1* and *BRG1* is not observed in a model of disseminated candidiasis. (A) The double homozygous *brg1*ΔΔ *efg1*ΔΔ shows a dramatic reduction in fungal burden relative to the wild type in a mouse model of oropharyngeal candidiasis. The log_10_-transformed fungal burden data (means and standard deviations) for each experiment were analyzed by one-way ANOVA followed by *post hoc* Student's *t* test to identify statistically significant differences between individual strains (*P* < 0.05). Strains that were statistically different from the WT are indicated with an asterisk. (B) The double heterozygous *efg1*Δ/*EFG1 brg1*Δ/*BRG1* mutant does not have a reduced kidney fungal burden relative to the WT or single heterozygous mutants in a mouse model of disseminated candidiasis. (C) The double heterozygous *efg1*Δ/*EFG1 brg1*Δ/*BRG1* mutant is as virulent as the WT and the single mutants in a mouse model of disseminated candidiasis based on Kaplan-Meier analysis of the disease progression curve shown.

To further confirm the genetic interaction between *EFG1* and *BRG1*, we generated the double homozygote mutant *brg1*ΔΔ *efg1*ΔΔ with the expectation that this strain would be even more severely affected than the *brg1*Δ/*BRG1 efg1*Δ/*EFG1* mutant. Consistent with that expectation, the tongues of mice infected with the double homozygous *brg1*ΔΔ *efg1*ΔΔ mutant were essentially sterile (Fig. 4A). Because the interaction between *EFG1* and *BRG1* was so significant in the murine model of OPC, we wondered if a similar interaction occurs during disseminated candidiasis. The *brg1*ΔΔ and *efg1*ΔΔ single mutants are both profoundly attenuated in the disseminated infection model, so examination of the double homozygous strain would not be informative because of a lack of dynamic range (14, 15). We therefore compared the double heterozygote (*brg1*Δ/*BRG1 efg1*Δ/*EFG1*) to the single heterozygotes (*brg1*Δ/*BRG1* and *efg1*Δ/*EFG1*) and WT in a model of disseminated candidiasis. As shown in Fig. 4B, the kidney fungal burden was slightly increased in animals infected with the *brg1*Δ/*BRG1 efg1*Δ/*EFG1* double heterozygous mutant, while the Kaplan-Meier curves for the four strains did not differ significantly (Fig. 4C). Thus, OPC infection is highly dependent on the genetic interaction between *EFG1* and *BRG1*, while this interaction is much less important during disseminated candidiasis.

### Expression of *TEC1* from a heterologous promoter restores the infectivity of the *brg1*Δ/*BRG1 efg1*Δ/*EFG1* mutant but not *bcr1*ΔΔ.

**FIG 5:**
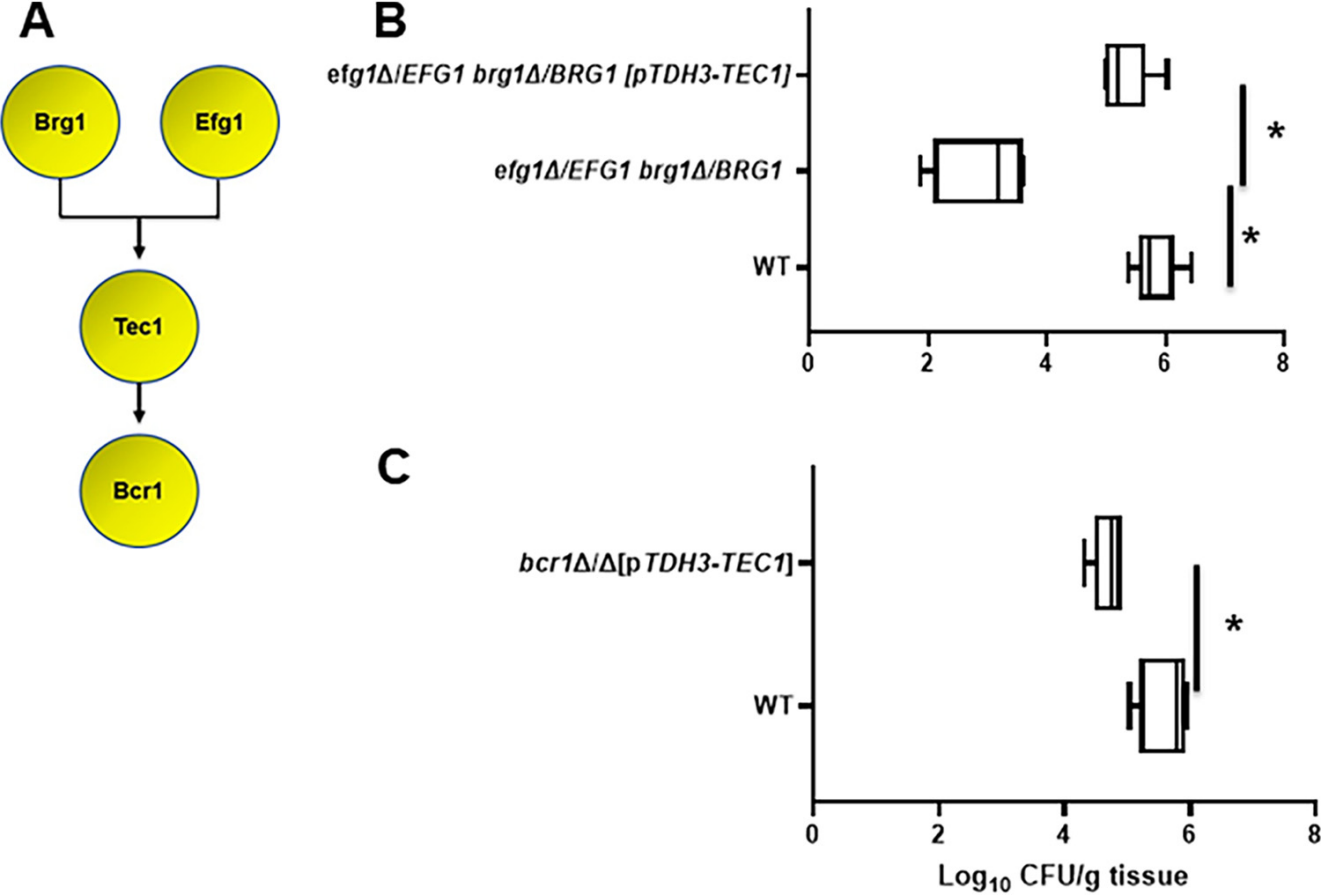
Epistasis analysis provides genetic support for a transcriptional circuit regulating infectivity in a mouse model of oropharyngeal candidiasis. (A) Diagram of proposed regulatory circuit based on *in vivo* genetic interaction data and known binding interactions *in vitro*. (B) Expression of *TEC1* from the *TDH3* promoter in the double heterozygous *efg1*Δ/*EFG1 brg1*Δ/*BRG1* mutant restores its infectivity in a mouse model of oropharyngeal candidiasis. The log_10_-transformed fungal burden data (means and standard deviation) for each experiment were analyzed by one-way ANOVA followed by *post hoc* Student's *t* test to identify statistically significant differences between individual strains (*P* < 0.05). (C) Expression of *TEC1* from the *TDH3* promoter in the homozygous *bcr1*ΔΔ mutant does not restore its infectivity in a mouse model of oropharyngeal candidiasis. *NS*, not significant.

During *in vitro* biofilm formation, the expression of *BCR1* is dependent on Tec1, while the expression of *TEC1* is partially dependent on Efg1 and Brg1 (9, 16). Based on these genetic interactions, one model for the transcriptional circuit regulating OPC is shown in Fig. 5A; the genetic interactions and the transcriptional circuit represent different relationships between the TFs. In this circuit, *TEC1* is a key node, and the model predicts that Brg1 and Efg1 play a critical role in the regulation of *TEC1* expression, which, in turn, regulates *BCR1*. To test this model, we took a genetic epistasis approach. If *TEC1* expression was critically altered in the *brg1*Δ/*BRG1 efg1*Δ/*EFG1* double mutant during OPC infection, we reasoned that placing *TEC1* under the control of a promoter that is independent of Brg1 and Efg1 would increase the infectivity of the double heterozygous mutant (11, 12). Consistent with that hypothesis, the *brg1*Δ/*BRG1 efg1*Δ/*EFG1* p*TDH3*-*TEC1* strain showed a 2.5-log_10_ increase in fungal burden relative to the parent double heterozygote (Fig. 5B). Previous *in vitro* data have shown that Tec1 regulates *BCR1* (16). Our data indicate that both *TEC1* and *BCR1* are required for full OPC infectivity. The *in vitro* experiment does not, however, establish that Bcr1 is an effector of Tec1 and, thus, the question of whether the function of Tec1 is dependent on Bcr1 remained open. To test the latter hypothesis, we placed *TEC1* under the *TDH3* promoter in the *bcr1*ΔΔ background. As shown in Fig. 5C, p*TDH3*-driven *TEC1* did not increase the fungal burden defect of *bcr1*ΔΔ. These data are consistent with the model that Bcr1 functions as a Tec1 effector.

### Transcription factor mutants with decreased infectivity in murine OPC undergo filamentation *in vivo*.

Five of the six TFs we examined have been shown to be required for filamentation under a wide range of *in vitro*-inducing conditions (9, 12); *BCR1* is the exception and is required for filamentation in some, but not all, clinical isolates (17) through interactions with other TFs under specific environmental conditions (12). Filamentation is required for biofilm formation, and previous work has indicated that some strains with reduced filamentation also have reduced infectivity in murine OPC. Thus, one potential mechanism for the reduced virulence of the mutants identified above is that they have reduced filamentation during infection.

**FIG 6:**
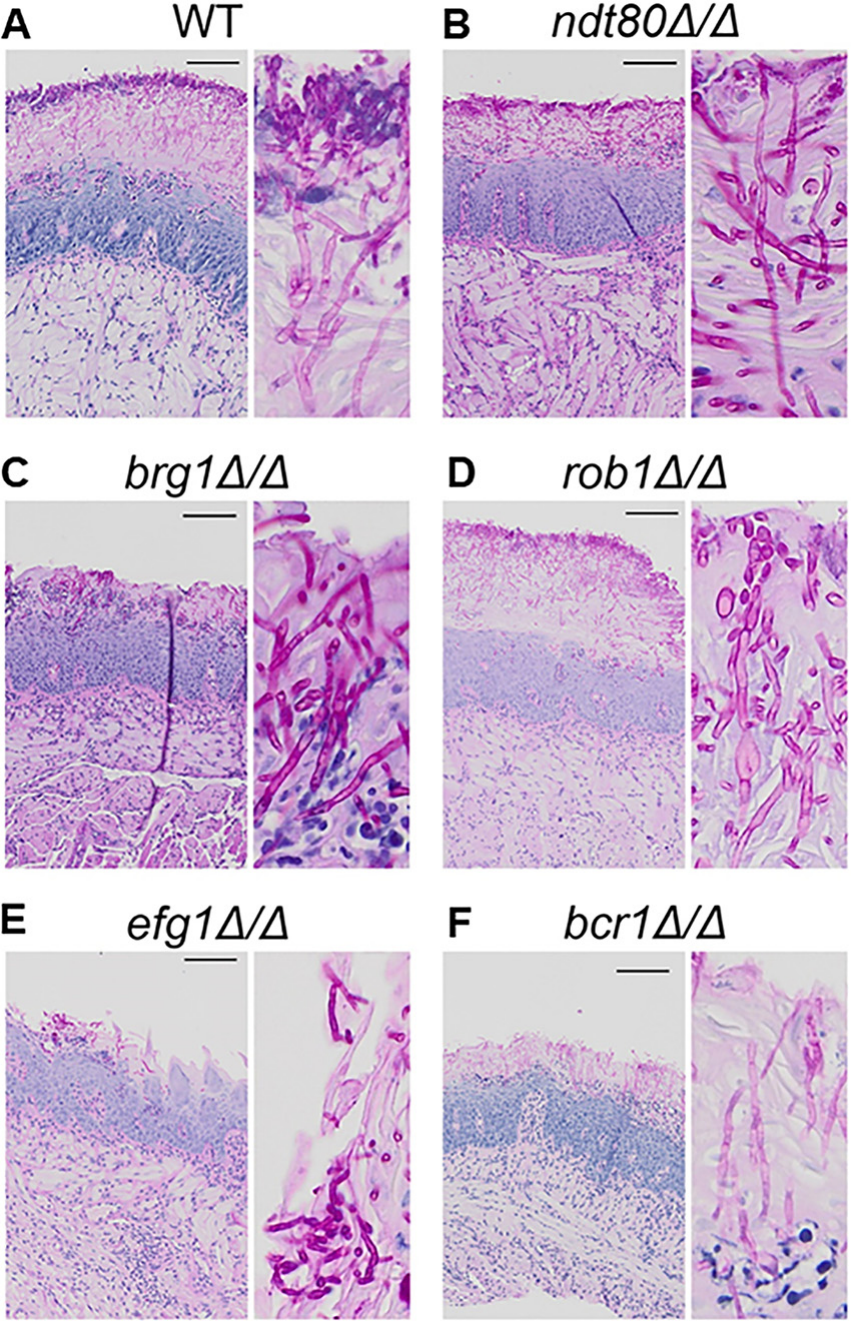
Transcription factor mutants with decreased infectivity undergo filamentation in tongue tissue. Histological sections of tongue tissue infected with the indicated strains were stained using the periodic acid-Schiff (PAS) reagent. C. albicans organisms stain fuchsia in lesions. Insets are provided to show morphology.

To test this hypothesis, we examined histologic sections of tongue infected with homozygous TF deletion mutants (Fig. 6). As expected, WT cells filament extensively within infected tongue tissue (Fig. 6A). Surprisingly, despite Ndt80, Brg1, Rob1, and Efg1 being involved in the yeast-to-hypha transition *in vitro*, none of these TF deletion mutants had significant defects in hyphal formation (Fig. 6B to E). For example, when grown under hypha-inducing conditions, *ndt80*ΔΔ mutants have a cell separation defect and form connected chains of pseudohypha-like cells (18). The tongue lesions formed by *ndt80*ΔΔ (Fig. 6B), however, contain cells that have filamentous characteristics that are indistinguishable from those observed in lesions infected with the WT (Fig. 6A). Brg1 is a well-characterized regulator of hyphal morphogenesis *in vitro* and plays a key role in the maintenance phase of hyphal elongation (15, 19). As with the *ndt80*ΔΔ mutants, the lesions on tongues infected with *brg1*ΔΔ mutants show robust filamentation (Fig. 6C).

Likewise, lesions induced by the *rob1*ΔΔ mutants show a predominance of hyphae (Fig. 6D) despite playing a role in filamentation during *in vitro* and *in vivo* biofilm formation on inanimate substrates (REF). *EFG1* is widely considered a master regulator of filamentation in C. albicans (14, 20). However, the fungal burden caused by *efg1*ΔΔ was not statistically different from the WT, and the lesions caused by infection with *efg1*ΔΔ mutants contained mainly filamentous fungal elements. Although close examination suggests the filaments of *efg1*ΔΔ mutant are not quite as robust as other strains (Fig. 6E), hyphal forms are present. *BCR1* does not affect filamentation in strains derived from SC5314, including the SN background used in this work (17), and, consistent with those observations, the *bcr1*ΔΔ mutant forms significant hyphae in the tissue (Fig. 6F). Thus, strains that have defects in establishment of infection (*bcr1*ΔΔ and *rob1*ΔΔ) and those that do not (*efg1*ΔΔ, *brg1*ΔΔ, and *ndt80*ΔΔ) form filaments in oral tissue. Thus, somewhat surprisingly, C. albicans TF mutants with decreased infectivity in the mouse OPC model retain the ability to undergo the yeast-to-hypha transition *in vivo* despite having significant filamentation defects *in vitro*.

### Saliva does not rescue the *in vitro* filamentation defects of transcription factor mutants required for OPC infection.

**FIG 7:**
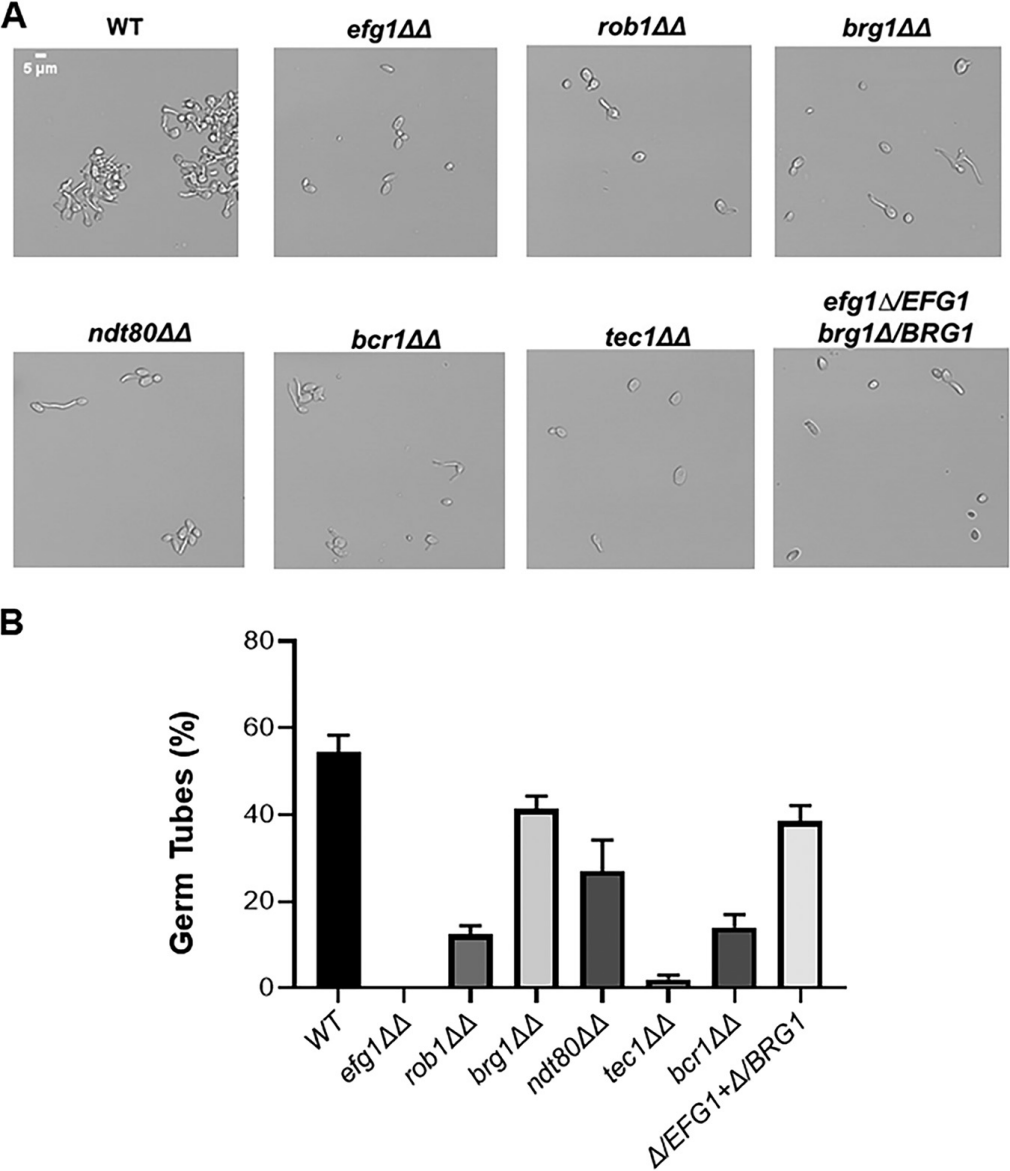
Biofilm-related transcription factor mutants are deficient for filamentation in human saliva. (A) The indicated strains were incubated in commercially sourced human saliva for 4 h at 37°C, fixed with formaldehyde, and imaged. The photomicrographs depict representative morphologies of the strains based on three biological replicates, with at least 100 cells evaluated in each replicate. (B) The bars indicate the percentage of germ tubes observed for each strain under the conditions described for panel A. The bars indicate means with error bars showing standard deviations. The statistical significance for changes from the WT were analyzed by ANOVA and Student's *t* test for individual comparisons, and statistical significance was defined as a *P* value of <0.05. Strains with a statistically significant change from the WT are indicated by an asterisk.

It is becoming increasingly apparent that the transcription factors required for filamentation vary with the specific environmental conditions. For example, *EFG1* is not universally required for C. albicans filamentation *in vitro*, because incubation under embedded conditions at room temperature (21) and coincubation with Streptococcus gordonii under synthetic oral biofilm conditions leads to filamentation (22). Furthermore, histology of oral tissue of gnotobiotic pigs infected with *efg1*ΔΔ mutants suggests that filaments are present under those conditions (23). With these observations in mind, it seemed possible that a component of the saliva might contribute to the filamentation of these strains. To test this, we incubated WT, *efg1*ΔΔ, *brg1*ΔΔ, *rob1*ΔΔ, *bcr1*ΔΔ, *rob1*ΔΔ, and *efg1*Δ/*EFG1 brg1*Δ/*BRG1* in commercially available whole human saliva at 37°C. After incubation for 4 h, germ tube formation is seen in 60% of wild-type cells (Fig. 7A). As shown in Fig. 7A and B, each mutant strain showed some level of reduced filamentation relative to the WT. Consistent with previous data, *EFG1*, *TEC1*, and *ROB1* were significantly affected. Prolonged incubation did not affect the comparisons, although the WT began to form lateral yeast, decreasing the ratio of yeast to filaments (data not shown). Somewhat surprisingly, the *brg1*ΔΔ mutant showed a modest phenotype while the *bcr1*ΔΔ strain displayed a 3-fold reduction in filamentation relative to the WT. Bcr1 typically does not affect filamentation in the SN background, but Huang et al. have shown that under some conditions and genetic backgrounds, Bcr1 plays a role in *in vitro* filamentation, particularly under biofilm-inducing conditions (17). Although not completely definitive, these data strongly suggest that the ability of these TF mutants to filament in the oral cavity is due to either interactions with tissue or perhaps commensal bacteria within the oral cavity.

### The Brg1-Efg1 interaction is required for oral epithelial cell invasion and is bypassed by constitutive expression of *TEC1*.

**FIG 8:**
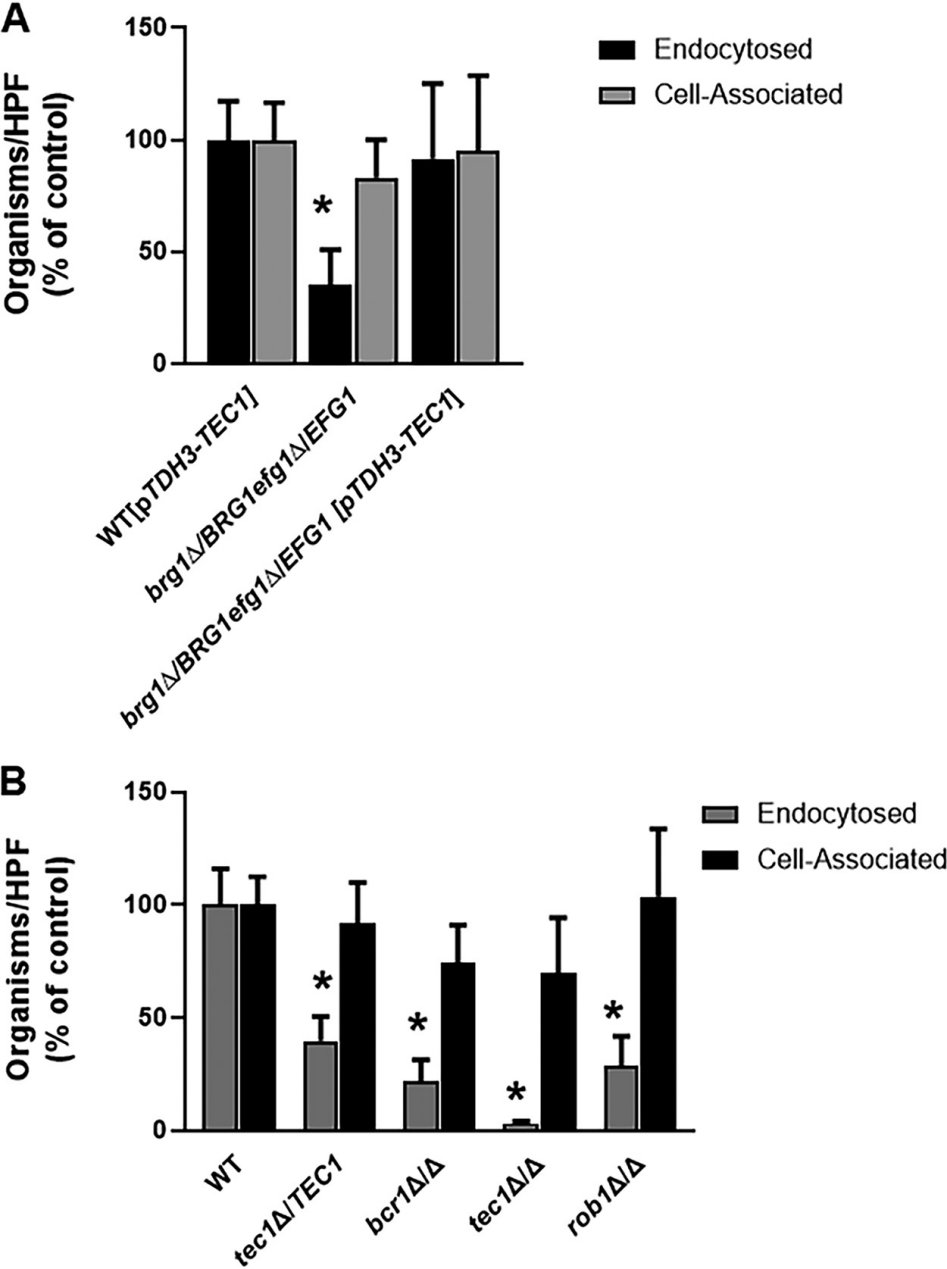
Transcription factor mutants with decreased infectivity in mouse model of oropharyngeal candidiasis have reduced induced endocytosis of oral epithelial cells. (A) The *efg1*Δ/*EFG1 brg1*Δ/*BRG1* double heterozygous mutant adheres to oral epithelial cells to an extent similar to that of the WT but has reduced endocytosis. Bars indicate means from two independent replicates, and error bars are the standard deviations. Groups were compared by one-way ANOVA followed by *post hoc* Student's *t* test to identify individual strains with statistically significant differences (*, *P* < 0.05). (B) Homozygous deletion mutants with reduced infectivity show deficits in oral epithelial cell endocytosis but not adherence.

To infect and damage oral tissue, C. albicans must adhere to and invade or be endocytosed by oral epithelial cells (24). To characterize which step of the process may be dependent on the Brg1-Efg1 interaction, we used a well-established *in vitro* model based on the OKF6/TERT-2 oral epithelial cell line (25). As shown in Fig. 8A, the *brg1*Δ/*BRG1 efg1*Δ/*EFG1* strain adheres to the epithelial cells as well as the WT, but the double heterozygous mutant has a significant defect in endocytosis. Consistent with the data from the OPC murine infection model, constitutive expression of *TEC1* from the *TDH3* promoter in the *brg1*Δ/*BRG1 efg1*Δ/*EFG1* mutant restores endocytosis. These data strongly suggest that Brg1 and Efg1 regulate genes critically important for oral epithelial cell endocytosis/invasion and that these transcription factors function upstream of *TEC1* during OPC.

Next, we examined the epithelial cell interactions of heterozygous and homozygous TF deletion mutants with decreased OPC fungal burden (Fig. 8B). Like the *brg1*Δ/*BRG1 efg1*Δ/*EFG1* double heterozygous mutant, the adherence of the haploinsufficient *tec1*Δ/*TEC1* cells to epithelial cells is similar to that of WT cells, but its rate of endocytosis is decreased significantly; indeed, the *tec1*Δ/*TEC1* mutant is similar to the *brg1*Δ/*BRG1 efg1*Δ/*EFG1* strain. The homozygous *tec1*ΔΔ strain shows the same pattern as the heterozygous *tec1*Δ/*TEC1* mutant, but the homozygous mutant is nearly completely unable to undergo endocytosis, clearly showing a gene dose effect. This phenotype is also displayed by both the *bcr1*ΔΔ and *rob1*ΔΔ mutants. Interestingly, loss of Tec1 leads to a more severe phenotype than loss of the downstream TF, Bcr1, suggesting that Tec1 has Bcr1-dependent and -independent effects on epithelial cell endocytosis.

## DISCUSSION

We have identified two new transcriptional regulators of OPC, *TEC1* and *ROB1*. Using a complex haploinsufficiency-based genetic interaction approach, we have also elucidated a regulatory circuit (Fig. 5B) that is required for C. albicans to infect oral tissue and that appears to mediate the ability of C. albicans to undergo induced endocytosis when attached to epithelial cells. Finally, we find that TF deletion mutants such as *efg1*ΔΔ and *brg1*ΔΔ undergo filamentation during oral infection despite being largely unable to filament under most *in vitro* conditions, suggesting that the transcriptional regulation of this process is dependent upon the specific environmental niche.

Our genetic interaction analysis focused on a set of TFs previously shown to be highly interconnected during the process of *in vitro* and *in vivo* biofilm formation (9, 11): *EFG1*, *TEC1*, *BCR1*, *BRG1*, *ROB1*, and *NDT80*. The pathobiology of OPC shares many key features with *in vitro* biofilm formation (6). Consistent with that model, previous studies have shown that, for example, the TF *BCR1* plays a critical role during *in vitro* biofilm formation, *in vivo* biofilm formation on oral dentures and intravascular catheters, and OPC in mouse models. Our results confirmed the role of *BCR1* in OPC (7, 8, 16) and found that *TEC1*, a regulator of *BCR1* expression *in vitro* (7, 9), is also required for full infectivity of C. albicans in this model. *ROB1* was the only other TF for which the homozygous deletion mutant showed reduced infectivity relative to WT reference strains. *In vitro* and during biofilm formation on in animate surfaces *in vivo*, all six of the TF mutants we examined showed profound defects, while during OPC only three of the mutants have significant phenotypes. This suggests that the functions of the interconnections between the TFs in this network are distinct during OPC.

One of the differences in our results compared to previous genetic analysis of OPC is that *efg1*ΔΔ mutants did not show a statistically significant effect (26). Previous studies reported by one of us found that *efg1*ΔΔ mutants were less able to establish infection during OPC (26). As shown in Fig. 1B, we observed significant variability in the fungal burden of mice infected with the *efg1*ΔΔ mutant. It is therefore possible that Efg1 affects OPC infectivity, but that would require larger sample sizes to confirm. It is also possible that *efg1*ΔΔ mutants are inherently variable in their phenotype for OPC, since that was also previously observed in the other experiment (26). It should also be mentioned that both the C. albicans and mouse strain backgrounds used in this work are different from that studied previously, SN versus CAI for C. albicans and CD-1 versus BALB/c for mice (26). As discussed below, we find that Efg1 does, in fact, play a role in OPC in conjunction with *BRG1*. Recent studies by Huang et al. have also shown that genetic circuits involving these very TFs can undergo significant diversification between different strain backgrounds in C. albicans (17). It is therefore also possible that the slightly diminished role of *EFG1* during OPC indicated by our single-gene deletion experiments results from subtle changes in network connections between different strain backgrounds, as described by Huang et al. (17).

Previous reports by Nobile et al. (9) and Glazier et al. (11) have firmly established that this set of TFs regulates the expression and function of each of the other five TFs. During *in vitro* biofilm formation, this TF network is an example of a highly fragile genetic network, because homozygous deletion mutants of any single TF are unable to form a biofilm (24). Further supporting the fragility of the *in vitro* biofilm network, single heterozygous deletion mutants have reduced biofilm formation (i.e., haploinsufficiency), and double heterozygous mutants have phenotypes that are similar to those of homozygous deletion mutants during biofilm formation (11). This is the opposite of a robust network where function is maintained in the face of multiple genetic disruptions (25). An example of a robust phenotype is the fact that very few TF deletion mutants show decreased growth in standard yeast media (27).

Robust networks are combinations of “or” statements in which function is maintained if this TF or that TF or this other TF is also functioning properly (28). Fragile networks, on the other hand, are combinations of “and” statements in which function is maintained only if this TF and that TF and this other TF are also functioning (28). Our analysis of this same network of TFs during OPC infection indicates that it is more robust than the fragile network characteristics displayed by the same mutants during *in vitro* biofilm formation. Supporting that conclusion are the following distinctions: (i) 3 of 6 homozygous deletion mutants have reduced OPC infectivity, while 6/6 have reduced *in vitro* biofilm formation; (ii) 1/6 heterozygous mutants showed haploinsufficiency during OPC, but 5/6 were haploinsufficient during *in vitro* biofilm formation; and (iii) 1/15 combinations of double heterozygotes showed a negative genetic interaction in OPC infection, while 6/15 displayed a negative genetic interaction during *in vitro* biofilm formation (11, 29).

A trivial explanation for the apparent robustness of this network during OPC would be that some of the component TFs have no role in the establishment of infection. However, our data argue against this explanation. First, five of the TFs (*EFG1*, *BRG1*, *TEC1*, *BCR1*, and *ROB1*) are required for OPC infection either based on single or double mutant phenotypes. Second, the remaining TF, *NDT80*, shows a positive interaction with *TEC1*, suggesting it functions in a buffering role. Interestingly, the interaction between *TEC1* and *NDT80* during OPC is the opposite of the strong, negative complex haploinsufficient interaction observed during *in vitro* biofilm formation (11). Overall, our genetic analysis of this network shows that its functional connections vary considerably between biofilm formation and OPC. The robustness of the latter may be because C. albicans*’* natural niche is the oral cavity of mammals and, thus, it is more highly adapted to colonization of this niche than inanimate surfaces.

The single negative complex haploinsufficent interaction that we identified, *EFG1-BRG1*, combined with our observation that *TEC1* and *BCR1* are critical for full infectivity during OPC and previous observations in the literature outlined above, allowed us to propose a potential genetic circuit (Fig. 5A). *In vitro* Tec1 regulates the expression of *BCR1* (16), and Brg1 and Efg1 are required for expression of *TEC1* (9); additionally, all three TFs bind each other’s promoters during *in vitro* biofilm formation (9). Hence, there is good literature precedent to support this model at the molecular level. Functionally, expression of *TEC1* from a promoter that is independent of *BRG1* and *EFG1* restored infectivity to the *brg1*Δ/*BRG1 efg1*Δ/*EFG1* strain, while it is not sufficient to do so with the *bcr1*ΔΔ mutant (Fig. 5B and C). Thus, our genetic epistasis data strongly support the model and suggest that Bcr1 regulates key effector molecules involved in establishing infection. Specifically, the overexpression of the Bcr1 target, *HWP1*, in a *bcr1*ΔΔ deletion mutant partially rescued OPC infection phenotypes (30). Our *in vitro* data regarding the expression of *HWP1* in mutants showing both positive and negative genetic interactions (Fig. 3) are consistent with its key role as a target of the regulatory circuit we have identified.

Although Bcr1 is critical for regulating adhesins required for the establishment of biofilms on abiotic surfaces (7, 9), we found that *bcr1*ΔΔ mutants as well as the other mutants in the circuit were able to bind to an oral epithelial cell line similarly to the wild type (Fig. 8A and B). Thus, the decreased infectivity of the mutants in this circuit does not appear to be due to reduced ability of the fungus to bind the epithelial surface. Instead, we found that the mutants showed a reduced ability to penetrate epithelial cells, a process mediated by induced endocytosis and/or direct penetration of the cells by hyphae (24). The latter mechanism appears to be an attractive explanation for some of our phenotypes, since 5/6 components of the network are known to regulate hypha formation *in vitro*.

We were, however, surprised to find that the homozygous deletion mutants with decreased OPC infectivity were able to undergo filamentation during infection based on histology of tongues infected with the mutants (Fig. 5). Thus, it seems unlikely that differences in hypha formation and, thus, direct epithelial cell penetration is the explanation for our results. Alternatively, it could be the case that the mutants fail to filament during the *in vitro* epithelial cell interaction studies and, thus, those studies could be confounded by differences in filamentation programs between *in vivo* and *ex vivo* conditions. It is also possible that differences in filamentation at an early stage of infection affect the ability of the mutants to establish infection or maintain infection. We have, however, previously shown that the *brg1*Δ/*BRG1 efg1*Δ/*EFG1* filaments similarly to the WT under tissue culture conditions (12), and, indeed, expression profiling of the double mutant under filament-inducing conditions focused on hypha-associated genes showed essentially no changes in expression relative to the WT (E. Do and A. P. Mitchell, unpublished results). Although a detailed understanding of the key outputs of this circuit during OPC awaits additional *in vivo* transcriptional profiling and characterization, it appears that the circuit regulates genes that are important for mediating induced endocytosis rather than the more highly studied adhesins.

Finally, our observations that *in vitro* regulators of hypha formation filament in the oral tissue represent an important finding. Most notable is the fact that mutants that lack two well-studied master regulators of filamentation, Efg1 and Brg1, form hyphae during oral infection. Similarly, Rob1 and Ndt80 are required for *in vitro* filamentation yet form filaments when infecting the oral tissue. As discussed above, there are conditions under which strains lacking *EFG1* will filament both *in vitro* and *in vivo* (23). Thus, the oral cavity may provide filamentation stimuli that bypass the requirement for Efg1 and other regulators of filamentation. Our data suggest that saliva is not responsible for this distinction (Fig. 7), implying interaction with tissue, oral microbes, or other factors specific to this niche are involved. Tec1 is also required for filamentation under a variety of *in vitro* conditions (29, 31) but has also been shown previously to form filaments in the tissue of mouse kidneys (31). In addition, we have recently found that the *tec1*ΔΔ mutant filaments in mouse tissue using an intravital imaging assay (32). Thus, our observations are part of a growing body of evidence that the transcriptional regulators required for C. albicans filamentation are highly contingent on the specific strain and particular host or environmental niche (17, 33).

In summary, a systematic *in vivo* genetic interaction analysis in a Candida albicans mammalian infection model has revealed network-level distinctions in how these TFs interact *in vivo* and during OPC. We have also identified a key transcriptional circuit that is required for infectivity and epithelial cell invasion. Future studies are needed to identify the key molecular outputs of this pathway and may identify novel mediators of C. albicans oral pathogenesis.

## MATERIALS AND METHODS

### Ethics statement.

All animal work was reviewed and approved by the Institutional Animal Care and Use Committee (IACUC) of both the University of Iowa Carver College of Medicine and the Lundquist Institute at Harbor-UCLA Medical Center.

### Strains and media.

The transcription factor (*EFG1*, *NDT80*, *BCR1*, *BRG1*, *TEC1*, and *ROB1*) heterozygous, homozygous, and double heterozygous deletion mutant strains are derived from the SN background and have been described previously (9, 11). These libraries were screened in the OPC model as described below. As such, the strains were auxotrophic for arginine. Independent isolates of TF mutants that showed decreased fungal burden in the OPC screening experiments were rendered prototrophic for arginine by transformation with a StuI-digested plasmid, pEXpARG, which integrated the *ARG4* gene into the *RSP10* site (34) before confirmation of the phenotypes in repeat OPC experiments. The *TDH3* promoter was integrated into the 5′ region of *TEC1* in *brg1*Δ/*BRG1 efg1*Δ/*EFG1* and *bcr1*ΔΔ by amplification of the promoter from plasmid pCJN542 (7) using a set of primers containing homology to the 5′ untranslated region and downstream of the *TEC1* ATG as previously described (11). Strains were routinely streaked on yeast-peptone–2% dextrose (YPD) plates from frozen stocks and incubated at 30°C. Prior to animal experiments or *in vitro* epithelial cell assays, the strains were grown overnight in liquid YPD with shaking at 30°C. The density of the culture was determined by hemocytometer and adjusted as indicated in the specific methods below.

### Mouse model of oropharyngeal candidiasis.

The pathogenicity of the C. albicans strains was tested in the immunosuppressed mouse model of OPC as previously described, with some modifications (35). Male outbred CD-1 mice were injected subcutaneously with cortisone acetate (300 mg/kg of body weight) on days −1, 1, and 3 relative to infection. On the day of infection, the animals were sedated with ketamine and xylazine, and a swab saturated with 10^6^ C. albicans cells per ml was placed sublingually for 75 min. After 5 days of infection, the mice were sacrificed and the tongues were harvested. The harvested tongues were either homogenized and plated for quantitative fungal burden or sectioned and processed for histology. The heterozygous and homozygous deletion mutants were tested in five animals per group. To limit the total number of mice, the double heterozygous mutants were first screened with three animals per strain to identify mutants with statistically significant defects (*P* < 0.05) or trends toward defects (*P* < 0.1) in the oral fungal burden. These mutants were then retested using five animals per group using strains that had been restored to arginine prototrophy. The log_10_-transformed fungal burden data for each experiment was analyzed by one-way analysis of variance (ANOVA) followed by *post hoc* Student's *t* test to identify statistically significant differences between individual strains (*P* < 0.05). Data reported in the figures are from the repeat experiments.

### Mouse model of disseminated candidiasis.

Assessment of fungal burden and disease progression in a model of systemic candidiasis was performed using fully immunocompetent mice (36). Female CD-1 outbred mice (6 to 8 weeks old; Envigo, Indianapolis, IN) were then inoculated by lateral tail vein injection with 10^6^ CFU of the indicated C. albicans strains. For determination of kidney fungal burden, mice were euthanized 72 h after inoculation. Kidneys were harvested, weighed, and homogenized in YPD. Tenfold dilutions of the homogenates were plated in duplicate on YPD and incubated overnight at 30°C. The kidney fungal burden then was calculated as number of CFU per gram of kidney tissue homogenized. For evaluation of the role of the C. albicans mutants in progression of disease, animals were inoculated as described above and monitored daily for clinical changes. Any animal that demonstrated symptoms of severe disease (extreme fur ruffling, abnormal posture, difficulty with ambulation, or failure to respond to surroundings) was euthanized immediately. Each experiment used 7 to 10 mice per group. Differences in the fungal burden were assessed using a one-way ANOVA followed by a *post hoc* Student's *t* test using the log_10_-transformed data for fungal burden to identify statistically significant differences between strains (*P* < 0.05). Disease progression was analyzed by Kaplan-Meier analysis and log rank (Mantel-Cox test, *P* < 0.05).

### RT-PCR analysis of *HWP1* expression under *in vitro* biofilm conditions.

The expression of *HWP1* in the indicated mutants was determined as described previously (9) using primers reported by Nobile et al. (9). Briefly, cells were harvested from 48-h biofilms generated in 6-well dishes at 37°C in YETS medium. After removal of supernatant and careful washing with phosphate-buffered saline (PBS), the adherent cells were scraped from the surface of the wells. RNA was isolated using the Qiagen RNA easy kit and reverse transcribed to cDNA using the Bio-Rad iScript kit. Quantitative PCR was performed as described previously (11), and *HWP1* expression was normalized to the *ACT1* gene and compared between mutants using the ΔΔ*C_T_* method. The data are presented as the means of 3 to 4 independent replicates performed in triplicate with standard errors of the means. Differences between mutants were analyzed by one-way ANOVA followed by Student's *t* test to assess significance of individual group differences (*P* < 0.05). We used the equation ε = [normalized phenotype of double mutant] – [expected phenotype of double mutant], where the expected phenotype is equal to the product of the normalized phenotypes of the individual single mutants; ε = 0 indicates no interaction and the genes function independently; ε > 1 indicates a positive or suppressive interaction; and ε < 1 indicates a negative or aggravating interaction.

### Characterization of C. albicans filamentation in human saliva.

Overnight cultures of the indicated strains were washed with PBS, resuspended to the initial volume, diluted 1:50 in commercially obtained human saliva, and incubated at 37°C for 4 h. The cells were fixed with 10% formaldehyde, and the morphologies characterized by light microscopy. The photomicrographs depict representative morphologies of the strains based on three biological replicates with at least 100 cells evaluated in each replicate. Data are presented as means with error bars showing standard deviations. The statistical significance for changes from the WT was analyzed by ANOVA and Student's *t* test for individual comparisons, and statistical significance was defined as a *P* value of <0.05.

### *In vitro* assay of C. albicans adhesion and induced endocytosis with oral epithelial cells.

To determine C. albicans adhesion to and invasion of oral epithelial cells, we used our established protocol (25). Briefly, OKF6/TERT-2 cells were grown to confluence on fibronectin-coated glass coverslips in 24-well tissue culture plates. The oral epithelial cells were infected with 2 × 10^5^ yeast-phase C. albicans cells per well. After incubation for 2.5 h, the cells were fixed with 3% paraformaldehyde and stained with an anti-*Candida* antibody (Meridian Life Science) conjugated with Alexa Fluor 588 (Thermo Fisher Scientific). After permeabilizing the epithelial cells with 0.05% Triton X-100 (Sigma-Aldrich), the cell-associated organisms were stained with the anti-*Candida* antibody conjugated with Alexa Fluor 488 (Thermo Fisher Scientific), and the coverslips were mounted inverted on microscope slides. The coverslips were viewed with an epifluorescence microscope, and the numbers of cell-associated and endocytosed organisms per high-power field was determined, counting at least 100 organisms per coverslip. Each experiment was performed three times in triplicate. The means and standard deviations for each strain were determined, and data were analyzed by one-way ANOVA followed by Dunnett’s *post hoc* test to identify individual strains with statistically significant differences (*P* < 0.05).
