# Supplementary figures and images for: Systematic Genetic Interaction Analysis Identifies a Transcription Factor Circuit Required for Oropharyngeal Candidiasis
Source: mBio. 2022 Jan 11;13(1):e03447-21. doi: 10.1128/mbio.03447-21 (PMC8749425; doi:10.1128/mbio.03447-21)

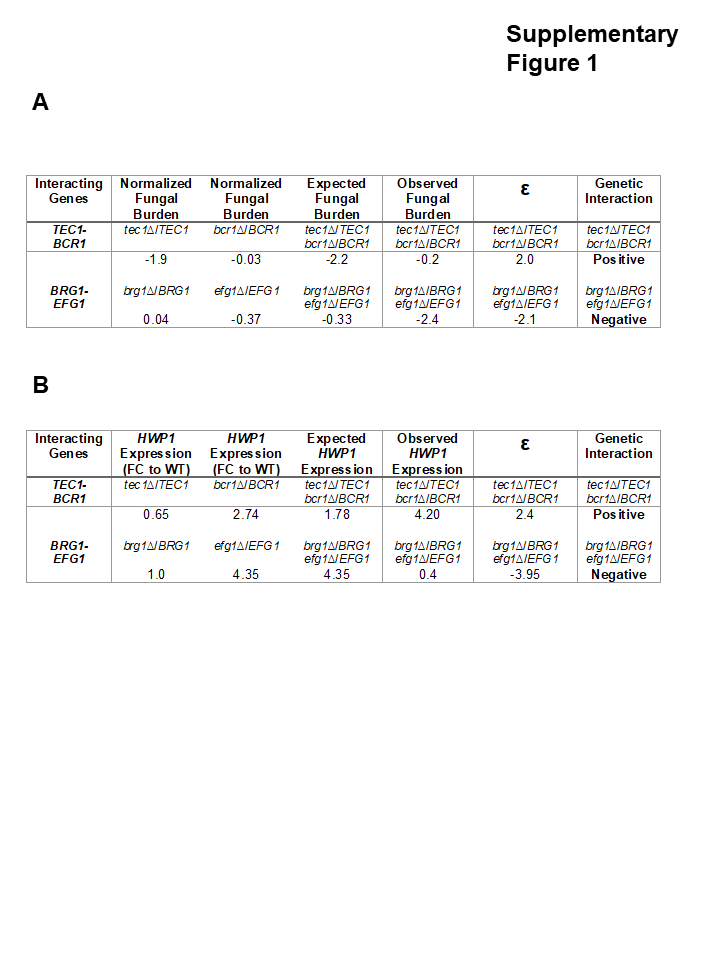

Supplement: FIG S1 [file mbio.03447-21-sf001.tif]
